# Parallel Digital Watermarking Process on Ultrasound Medical Images in Multicores Environment

**DOI:** 10.1155/2016/9583727

**Published:** 2016-02-11

**Authors:** Hui Liang Khor, Siau-Chuin Liew, Jasni Mohd. Zain

**Affiliations:** Faculty of Computer Systems and Software Engineering, University Malaysia Pahang, Lebuhraya Tun Razak, Gambang, 26300 Kuantan, Pahang Darul Makmur, Malaysia

## Abstract

With the advancement of technology in communication network, it facilitated digital medical images transmitted to healthcare professionals via internal network or public network (e.g., Internet), but it also exposes the transmitted digital medical images to the security threats, such as images tampering or inserting false data in the images, which may cause an inaccurate diagnosis and treatment. Medical image distortion is not to be tolerated for diagnosis purposes; thus a digital watermarking on medical image is introduced. So far most of the watermarking research has been done on single frame medical image which is impractical in the real environment. In this paper, a digital watermarking on multiframes medical images is proposed. In order to speed up multiframes watermarking processing time, a parallel watermarking processing on medical images processing by utilizing multicores technology is introduced. An experiment result has shown that elapsed time on parallel watermarking processing is much shorter than sequential watermarking processing.

## 1. Introduction

The technological advancement in communication network has facilitated healthcare professionals across the world in accessing electronic medical records, such as medical images, and obtaining second opinions for high-quality diagnosis. As a consequence, medical images are exposed to security threat such as tampering of images, which may lead to wrong diagnosis and treatment [1]; thus a digital watermarking on medical image is introduced. So far most of the watermarking research has been done on single frame medical image which is impractical in the real environment, where most of the ultrasound medical images consist of multiframes; thus a digital watermarking on multiframes medical images is proposed. Watermarking could be applied on ultrasound medical images frame by frame sequentially but it would be time consuming for a large dataset; thus a parallel environment is necessary for speeding up multiframes watermarking process by utilizing multicore technology. The performance improvements gained by the use of a multicore processor largely depended on the software algorithms used and their implementation. According to Amdahl's law, the possible gains are only limited on software portion that can be run in parallel simultaneously on multiple cores. Our work explores efficient parallel implementations of the digital watermarking scheme in multicore environment.

## 2. Theory: Concept of Digital Watermarking

Digital watermarking is the technology that imperceptibly modifies original data and embeds them directly into image. Digital watermarking in general is comprised of three major components [2]:

1. Watermark generator: a desired watermark(s) is generated for particular applications, which are optionally dependent on some keys.
2. Watermark embedder: watermark(s) are embedded into the object, sometimes based on an embedding key.
3. Watermark detector: detecting the existence of some predefined watermark in the object. It is sometimes desirable to extract a message as well.

The purpose of medical image security is to maintain privacy of the patient information in the image and to assure data integrity that prevents the image from tampering [3]. Watermarking can be used in medical images to prevent unauthorized modification by authenticating the content of the image. Tamper localization capable watermarking scheme can detect and locate modification of pixel values on the image [4]. The tampered area can be recovered by retrieving the original pixel values that were stored on the image itself as a watermark. Tamper localization is useful for deducing the motive of the tampering and whether any modification is legitimate [5]. Liew and Zain proposed a reversible watermarking scheme (TALLOR watermarking scheme) by dividing image into ROI (Region of Interest) and RONI (Region of Noninterest) [6]. ROI is the significant part of the medical images that is used by doctors to diagnose the patient, and RONI is the area outside the ROI. Watermarking for tamper detection and recovery is done in the ROI area based on Jasni's scheme [7]. The original Least Significant Digits (LSBs) that are removed in watermark embedding process are stored in RONI after compression. The stored LSBs later can be used to restore the image to its original bits value so the watermarking scheme can be reversible [8]. The research was conducted on a single frame of ultrasound which is impractical in the real world; thus a digital watermarking on multiframes medical images is proposed. In order to speed up multiframes watermarking processing time, a parallel computation in multicores watermarking processing on medical images is introduced.

## 3. Theory: Parallel Computing in Multicores

In recent years there has been a surge of interest in running application in parallel to take advantage of multiprocessor and multicore systems. Developments in microprocessor technologies have resulted in most processors having multiple computing cores in a single chip [9]. Parallel computing is a concept of performing tasks simultaneously by partitioning a large and complex problem into smaller tasks and solving each of them concurrently. There are two forms of parallelism: task parallelism and data parallelism (as shown in Table 1).

Data parallelism emphasizes the distributed (parallelized) nature of the data, as opposed to the processing (task parallelism) [10]. Data parallelism is adopted in this experiment since each processor performs the same code (watermarking code) on different pieces of distributed ultrasound frames.

Optimal speedup from parallelization should be linear if the number of processing elements is inversely proportional to its run time. However, not many parallel algorithms achieve optimal speedup. Most of them have a near-linear speedup for small numbers of processing elements, which flattens out into a constant value for large numbers of processing elements [11].

According to Amdahl's law, the overall speedup from parallelization would be restricted by a small portion of the program which cannot be parallelized. A large and complex program usually consists of several parallelizable parts and several nonparallelizable (sequential) parts. If *α* is the fraction of running time a program spends on nonparallelizable parts [12], then

(1) $$\begin{array}{c} \underset{P\to \infty }{\lim}\frac{1}{\left(1-\alpha \right)/P+\alpha }=\frac{1}{\alpha }. \end{array}$$

It is the maximum speedup with parallelization of the program, with *P* being the number of processors used. If the sequential portion of a program accounts for 10% of the runtime (*α* = 0.1), then a 10x speedup will be the maximum, regardless of how many processors are added. This generates an upper limit on the usefulness of adding more parallel execution units [13].

Gustafson's law is another law in computing, closely related to Amdahl's law [9]. It states that the speedup with *P* processors is

(2) $$\begin{array}{c} S\left(\;P\;\right)=P-\alpha \left(\;P-1\;\right)=\alpha +P\left(\;1-\alpha \;\right). \end{array}$$

Amdahl's law assumes that the total amount of work to be done in parallel is also independent of the number of processors, whereas Gustafson's law assumes that the total amount of work to be done in parallel varies linearly with the number of processors [12].

Applications are often classified based on the frequency of synchronization and communication needs between their subtasks. Fine-grained parallelism is where an application has a high rate of communication among subtasks; coarse-grained parallelism is where an application does not communicate many times per second, and it is embarrassingly parallel if an application seldom or never has to communicate. Embarrassingly parallel applications are considered the easiest to parallelize [14]. In the embarrassingly parallel problems, speedup factors could be achieved near the number of cores, or even more if the problem is partitioned enough to fit within each core's cache(s), avoiding use of much slower main system memory.

## 4. Parallel Computing with MATLAB

### 4.1. How Parallel Computing Runs a Job

**Figure 1:**
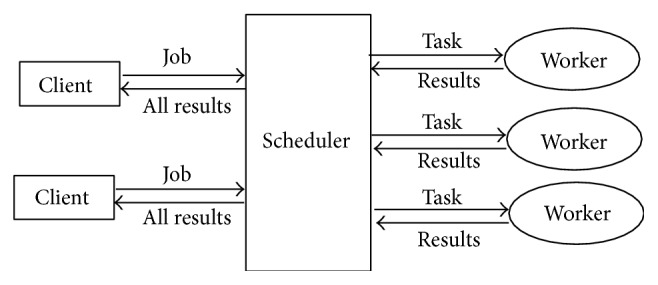
Interactions of Parallel Computing Session.

The* MATLAB job scheduler* (MJS) is the process that coordinates the jobs execution and their tasks evaluation. The MJS can be run on any machine on the network. The MJS runs the submitted jobs in queue order, unless any jobs in its queue are promoted, demoted, cancelled, or deleted. MJS assigns task from the running job to each worker for execution and fetches result from workers upon the task completion. The cycle is repeated with another task. When all tasks for a running job have been assigned to workers, the MJS starts running the next job on the next available worker. Tasks were executed simultaneously by all workers in order to speed up execution of large MATLAB jobs. The MJS then returns the results of all the tasks in the job to the client session (as shown in Figure 1) [15]. In this research, client and MJS are located in a single computer where a client (end user) has sent a job (a stack of ultrasound medical images) into MJS where it segregates the ultrasound medical images into multiple tasks (subset of ultrasound medical images frames) into workers (cores) and executes watermarking process in parallel. For example, in quad cores computer, MJS will segregate 15 frames of ultrasound medical images into 4,4,4,3 frames to each core/worker, respectively. The details process will be discussed in Section 5.

### 4.2. Life Cycle of a Job

**Figure 2:**
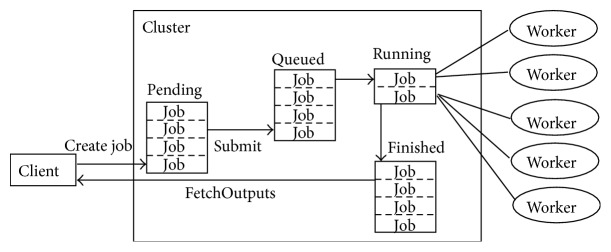
Stage of a job.

Job progresses through a number of stages upon its creation. In the MJS (or other schedulers), each stage of a job is categorized by their state, such as pending, queued, running, or finished (refer to Table 2).  Figure 2 illustrates the stages in the life cycle of a job. Functions used in job management are createJob, submit, and fetchOutputs [14].

Table 2 describes each stage in the life cycle of a job [15].

## 5. Research Methodology: Sequential and Parallel Watermarking Embedding and Authentication Process

**Figure 3:**
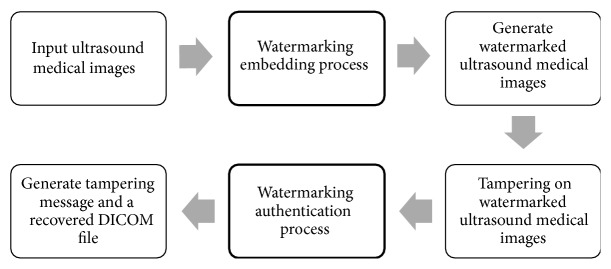
Watermarking process flow on a single frame of ultrasound medical image.

In watermarking process on a single frame of ultrasound medical image, watermark is embedded into ultrasound medical images and becomes an input file for watermarking authentication process (as shown in Figure 3). The purpose of authentication process is to localize and recover the tamper region in medical images. In other words, the prerequisite of watermarking authentication process is watermarked ultrasound medical images, in which it is the output file generated by watermarking embedding process. Process watermarking frame by frame sequentially was time consuming; thus parallel computing on multicores was introduced to solve the problem concern. This is accomplished by dividing ultrasounds frames into tasks to each core, respectively, and performing watermarking process simultaneously with others.

Two modes of watermarking process in multiframes will be developed and compared; there is sequential (by using for loop) versus parallel watermarking process (watermarking on multicores); the purpose is to prove that the parallel watermarking will have a significant improvement on elapsed time. In both sequential watermarking embedding and authentication process, watermarking is processed frame by frame sequentially by using a control loop. Therefore the elapsed time is proportional to the number of frames processed. The elapsed time could be reduced by using parallel computing on multicore processing technique and this technique enables ultrasound frames to be divided and distributed to multicore for parallel watermarking processing.

### 5.1. Sequential Watermarking Process

**Figure 4:**
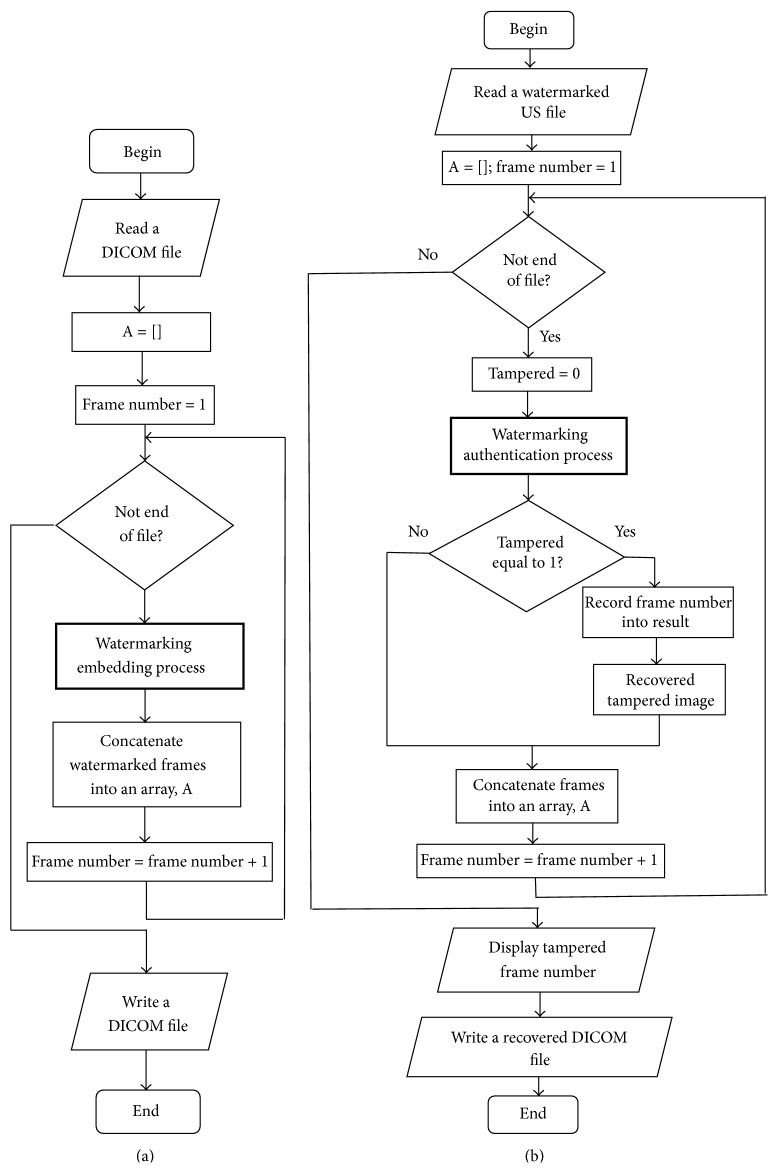
Program flow chart for (a) sequential watermarking embedding process and (b) sequential watermarking authentication process.

Sequential watermarking embedding and authentication process are sharing a common framework, where ultrasound medical images in DICOM format are read and perform watermarking process frame by frame sequentially by using a for loop. Processed frames will then concatenate into a variable named as “A” which will convert into DICOM format at the end of the watermarking process. The relationship between both processes is that the output file of watermarking embedding process is the input file for watermarking authentication process. The difference between them is that authentication process has an additional step in identifying the tampered frames; if it is 0, it means nontampered; else it is tampered and then image recovery is performed and tampered frame number is recorded and will be displayed upon the completion of watermarking process (as demonstrated in Figure 4). The main algorithms of parallel watermarking process are dividing volumetric ultrasound medical images and distributing them into a number of cores and executing sequential watermarking processes on each core in parallel; therefore a successful sequential watermarking process is a prerequisite in parallel watermarking process. The details of parallel watermarking process will be discussed in Section 5.2.

### 5.2. Parallel Watermarking Process

**Figure 5:**
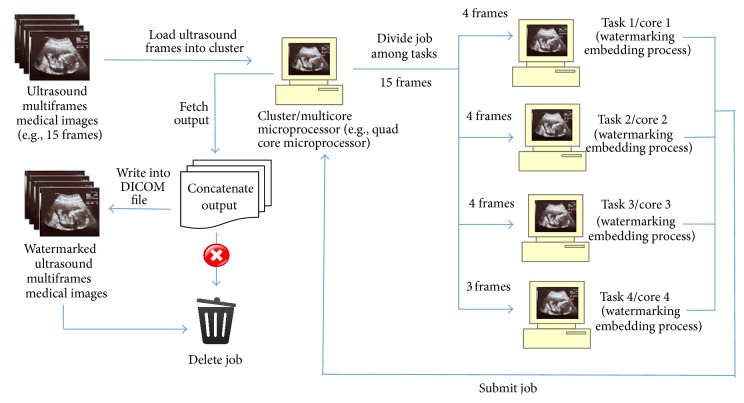
Parallel watermarking embedding process.

**Figure 6:**
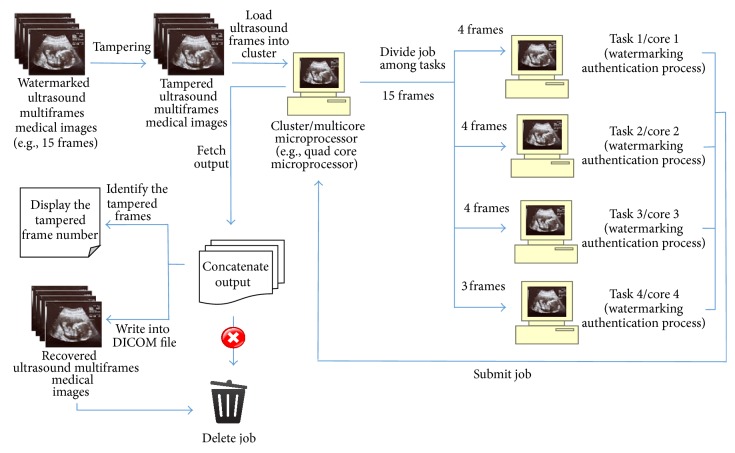
Parallel watermarking authentication process.

In parallel watermarking process (as illustrated in Figures 5 and 6), ultrasound multiframes medical images were loaded into a quad core microprocessor/cluster and create a job on the scheduler; the job is then divided into tasks according to the number of cores in the microprocessor.

**Figure 7:**
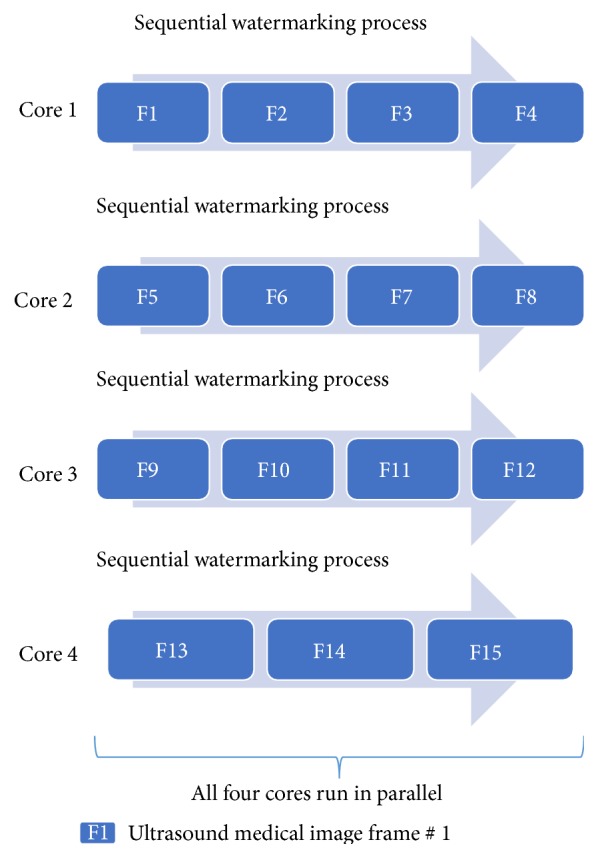
Inside look of parallel watermarking process that runs on 15 frames of ultrasound medical images.

The code implemented enables cluster to autodetect the number of cores available in the processor; if the processor used is a quad core, then the job is divided into 4 tasks, where ultrasound frames are equally divided by 4; for example, if the total number of ultrasound frames is 30, then it will be divided into 8,8,7,7 frames, and if the total number of frames is 15, then it will divided into 4,4,4,3 frames. Those divided frames will then distribute to 4 cores, respectively. In each core, watermarking process is carried out sequentially on the divided frames and at the same time it runs concurrently with other cores (as illustrated in Figure 7).

It is important to ensure that the generated frames output is in order after parallel watermarking process; therefore the frame number has to be assigned and keep track before the frames are sent to the cores, respectively. Upon the tasks completion, tasks were reassembled into a job, whereby all the frames will concatenate into an array and submit back to the cluster. The result is retrieved from all the tasks in the job with the function fetch output. All the frames will concatenate according to the frame number order and write into a DICOM file. A job is deleted on two circumstances:

1. When the scheduler encounters an error.
2. When the job is finished.

Both parallel watermarking embedding and authentication process have a similar process flow as described above, except the process applied on each task, input and output files as listed in Table 3. The input file of authentication process is the tampered output file of embedding process.

## 6. Experimental Design and Set-Up

TALLOR watermarking scheme, developed by Liew and Zain [4], will be executed in sequential and parallel modes. The elapsed time obtained from both modes will be compared and speedup factor of parallel relative to sequential watermarking process will be measured. It is to verify the efficiency of parallel framework.

Three important performance metrics were studied. These are

1. imperceptibility: testing the quality of medical images in terms of invisibility of watermarking in multiframes environment;
2. elapsed time: the time taken to perform watermarking embedding and authentication process on medical images in multiframes environment;
3. robustness to tampering: testing the effectiveness and efficiency of the tamper detection, localization, and recovery function in multiframes environment.

The evaluation was performed by running MATLAB (The Mathworks, Inc., Natick, MA, USA) program on a laptop with quad core CPU of Intel® Core*™* i7-3630QM CPU @ 2.4 GHz, 2401 MHz, 4 Core(s), 8 Logical Processor(s), and RAM of 8 GB. Three samples of ultrasound medical images in DICOM format were used to test the system (as shown in Table 4).

## 7. Experimental Result

### 7.1. Imperceptibility

The perceptibility of a watermarked image can be judged according to its fidelity and quality. Fidelity measures the similarity between images before and after watermarking [16]. A high fidelity means that watermarked image is very similar to the original image. The mean-squared-error (MSE) and peak signal-to-noise ratio (PSNR) were calculated by comparing the watermarked image and original image. Watermarked images may bear visible or invisible distortion due to the embedding process. One way to quantify distortion is the mean-square error. If **I**_*i*_′ is a vector of *n* predictions, and **I**_*i*_ is the vector of observed values corresponding to the inputs to the function which generated the predictions, then the MSE of the predictor can be estimated by

(3) $$\begin{array}{c} MSE=\frac{1}{n}\overset{n}{\underset{i}{\sum }}{\left(\;I_{i}^{\prime }-I_{i}\;\right)}^{2}. \end{array}$$

MSE is the average term by term difference between the original image, **I**, and the watermarked image, **I**′. If **I** and **I**′ are identical, then MSE(**I**′, **I**) = 0. A related distortion measure is the peak signal-to-noise ratio (PSNR), measured in decibels (dB). The problem with mean-square error is that it depends strongly on the image intensity scaling while PSNR rectifies this problem by scaling the mean-square error according to the image range [17]. PSNR is defined as follows:

(4) $$\begin{array}{c} PSNR\left(\;dB\;\right)=10\;\log_{10}\frac{\max I^{2}}{{MSE}^{\prime }}, \end{array}$$

where max⁡**I** is the peak value of the original image. If the signals are identical, then PSNR is equal to infinity. A high PSNR represents a high fidelity of a watermarked image. In this thesis, PSNR is used as a measurement for image fidelity. A high-quality watermarked image does not have any obvious noticeable distortion caused by the watermark embedding process. The assessment of quality is usually evaluated by human observers and is influenced by personal preferences which are subjective in nature.

Three different sets of ultrasound medical images which contain thirty and fifteen frames have been watermarked in two different ways: (1) sequentially and (2) parallel. It is important to ensure that the quality and fidelity of images were not affected by the way watermarking embedding process performed. Both sequential and parallel watermarking embedding processes have produced the same MSE and PSNR result for each frame as indicated in Table 5 except some negligible differences in the highlighted areas. This means that the operation either in sequential or in parallel mode does not affect the image quality and its fidelity. The PSNR values reflect the medical image integrity and high PSNR values indicate lesser distortion on medical image after watermarking process. PSNR value reflected medical image fidelity; ideally the watermarked medical image should be visually indistinguishable as original image. The PSNR values are calculated for all images ranging within 48.29~48.74 dB, which are within the acceptable range for diagnosis purposes and it has achieved imperceptibility as shown in Figure 9 where the images before and after watermarking embedding process are visually indistinguishable as the original images.

### 7.2. Elapsed Time

**Figure 8:**
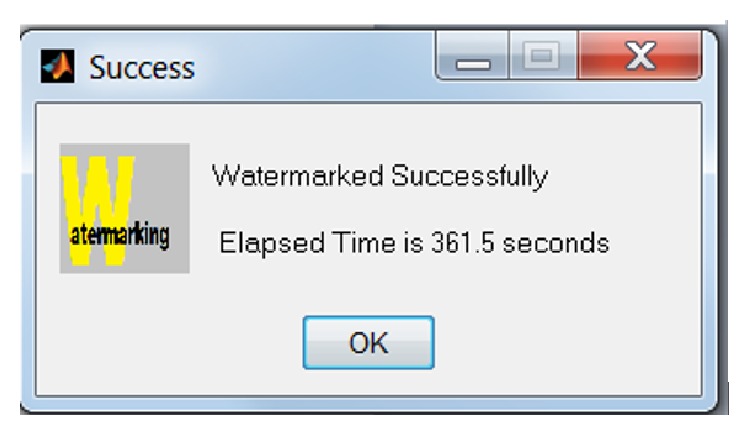
Message displayed upon the completion of sequential watermarking embedding process.

**Figure 9:**
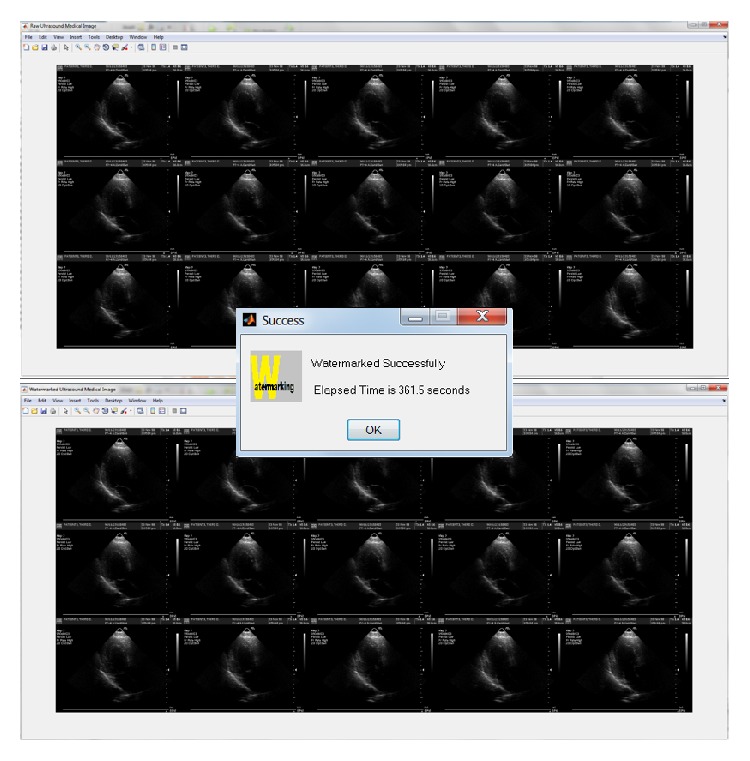
The output of raw and watermarked ultrasound medical images after a sequential watermarking embedding process.

Elapsed time is the time taken to perform watermarking embedding (Figure 8) and authentication process (Figure 12) on medical images in multiframes environment. This section is to test the speedup factor in parallel mode as compared to sequential mode in watermarking process. The formula of speedup factor is defined as follows:

(5) $$\begin{array}{c} Speed\;\;up=\frac{Elapsed\;\;time\;\;in\;\;sequnetial\;\;mode}{Elapsed\;\;time\;\;in\;\;parallel\;\;mode}. \end{array}$$

Speedup factor is to measure the speed of parallel mode by factors relative to sequential mode. For example, if the speedup is 3, this means parallel process is three times faster than sequential mode.

#### 7.2.1. Watermarking Embedding Process

**Figure 10:**
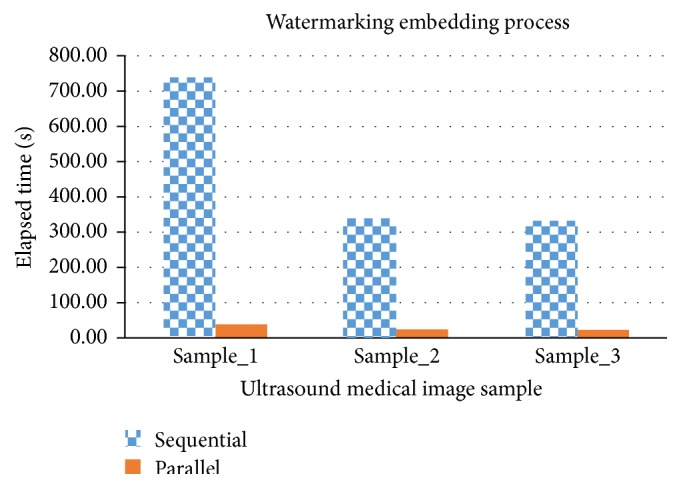
Sequential versus parallel watermarking embedding process in elapsed time.

Table 6 shows that watermarking embedding process in parallel has achieved a significant speedup (14.13~19.29) relative to sequential process. In Figure 10, the elapsed time in sequential watermarking embedding process on sample_2 and sample_3 is similar but increases by double in sample_1; this means that the elapsed time in sequential process is proportional to the number of frames, whereas in parallel watermarking embedding process, the elapsed time results are consistent despite the number of frames processed. In conclusion, the number of frames does not have much impact on the parallel process as compared to sequential process. The watermarking embedding scheme is pixel oriented, which means different ultrasound sample with the same frame size will produce a similar result.

The elapsed time is actually depending on many factors such as processor type, clock rate, memory speeds and use of memory catches, location of code in memory, compiler efficiency, and compiler optimization technique.

#### 7.2.2. Watermarking Authentication Process

Different to watermarking embedding process, watermarking authentication process is to verify whether there is any tampering that occurred in the watermarked ultrasound medical images and then recovered the tampered frame to its original state. In Table 7, three watermarked ultrasound medical images have been fully tampered and the speedup factor ranges within 2.92~3.64. The speedup factor is lesser as compared to watermarking embedding process. This is because, firstly, watermarking authentication algorithm has more if-else branches as compared to watermarking embedding algorithm and, secondly, watermarking authentication process needs to return two results (a string of tampered frames and concatenated ultrasound frames), whereas watermarking embedding process just returns one result, that is, concatenated frames of watermarked ultrasound medical images.

**Figure 11:**
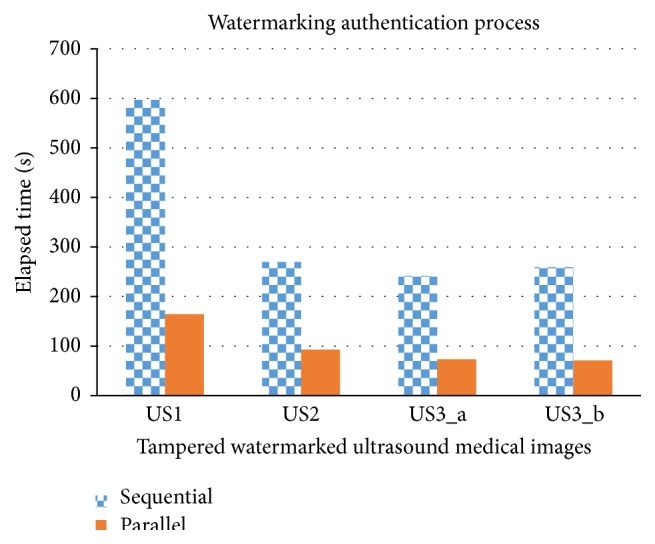
Sequential versus parallel watermarking authentication process in elapsed time.

In watermarking authentication process also is pixel oriented; therefore the different sources have shown a little impact on elapsed time. It could be observed in Figure 11 that watermarking authentication process in sequential mode is proportional to frame size but it is not the case in parallel mode; the elapsed time does not have many changes in parallel mode at different frame size.

#### 7.2.3. Overall Performance of Watermarking Embedding and Authentication Process

The whole package of watermarking process is involving two steps: (1) watermarking embedding process and (2) watermarking authentication process. Therefore it is necessary to test the overall elapsed time involved in both processes. The high speedup in watermarking embedding process is compromised by the low speedup in watermarking authentication process.  Table 8 has shown that the overall speedup factor for three ultrasound medical images samples ranges within 5.21~6.60.

### 7.3. Robustness to Tampering

In order to demonstrate the tamper localization function in detecting forgery, counterfeited images were created by manually modifying the pixel values in the watermarked images using image processing software—ImageJ 1.46r.  Figure 13 shows an example of tampering on three frames (frames numbers 2, 4, and 6) in ultrasound watermarked medical images.

**Figure 12:**
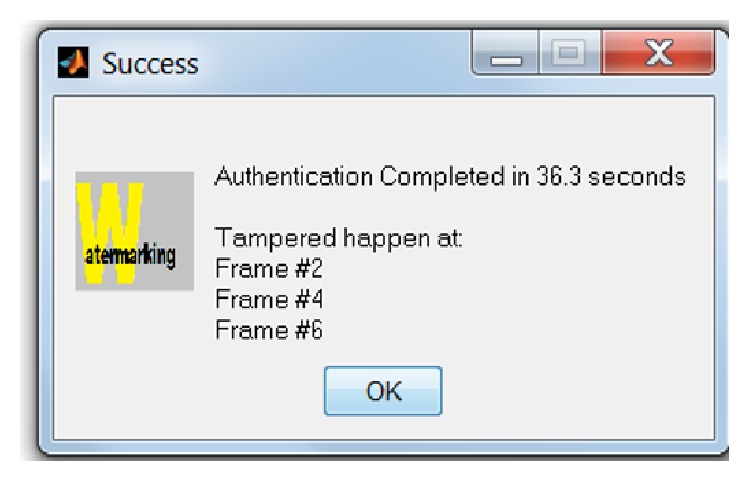
A message displayed after watermarking authentication process has been completed.

**Figure 13:**
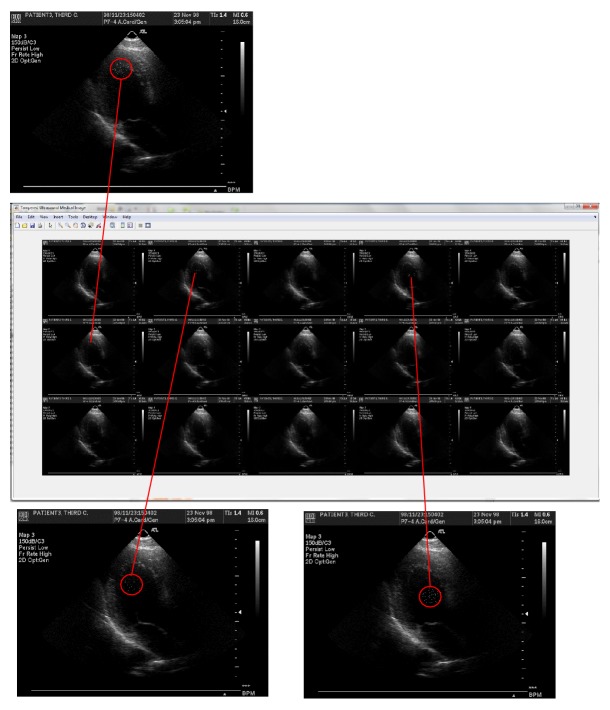
Ultrasound medical images tampered on frame numbers 2, 4, and 6.

**Figure 14:**
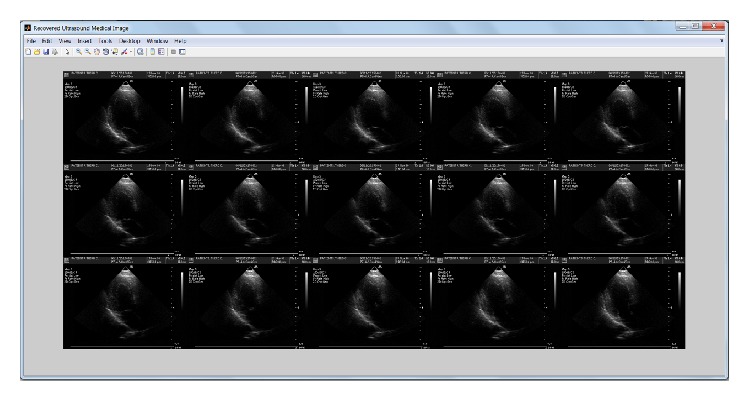
Recovered ultrasound medical images after watermarking authentication process.

**Figure 15:**
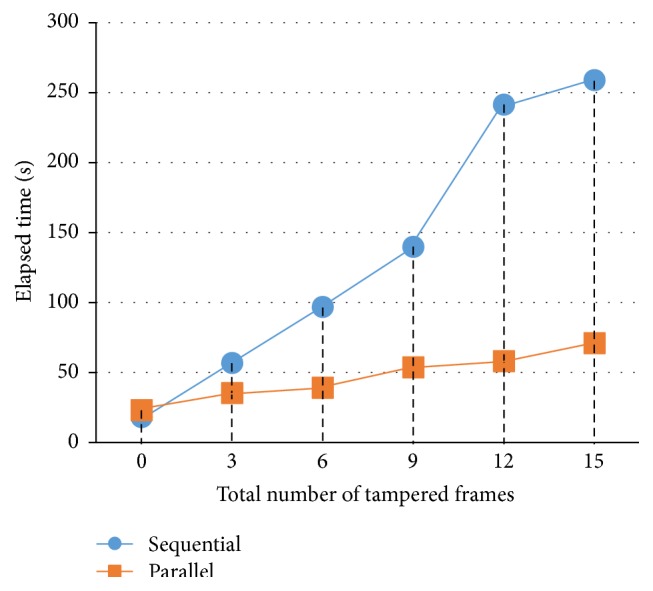
Comparison of elapsed time between sequential and parallel watermarking authentication process on different number of tampered frames.

Different set of tampered watermarked ultrasound medical images has been used to test the effectiveness and efficiency of the tamper detection, localization, and recovery function in multiframes environment (as shown in Table 9). The function's effectiveness was measured by checking whether it could detect the tampered frame number in multiframes environment (as shown in Figures 12 and 13) and is able to recover to its original form (as shown in Figure 14). The function's efficiency was measured by comparing the elapsed time taken for both sequential and parallel watermarking authentication process (as shown in Figure 15). Both effectiveness and efficiency testing were performed while testing the function's robustness to tampering.

For tampered free watermarked ultrasound medical images, the initial setup of parallel authentication process such as determining number of cores and dividing and distributing frames to each core was time consuming; therefore it takes longer processing time than sequential version.

For both sequential and parallel watermarking authentication process, the elapsed time is proportional to the total number of tampered frames. Relative to sequential watermarking authentication, the efficiency of parallel watermarking authentication showed remarkably when there are more tampered frames. In summary, the elapsed time for parallel watermarking authentication process is varied based on the total number of tampered frames in ultrasound medical images.

With 100% tampered watermarked ultrasound medical images, the efficiency of parallel version can be calculated based on elapsed time, such as 259.1/71 = 3.65, which means parallel version can perform 3.65 times faster than sequential version. In other words, sequential watermarking authentication has performed a job all by itself, whereas parallel watermarking authentication has delegated a job to four workers/cores, in which parallel version has performed approximately 4 times faster than sequential version. In conclusion, the speedup factor was approximate to the number of cores since there is little communication between subtasks.

**Figure 16:**
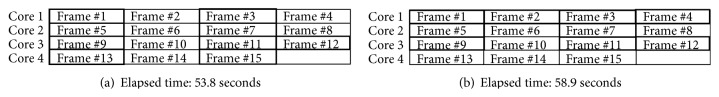
The elapsed time taken in different order of tampered frames organization during parallel watermarking authentication process.

The frame order of tampered frame also is a factor that affects the performance in parallel process (as shown in Figure 16). The black rectangle boxes with bold font represent a tampered frame. Both (a) and (b) have distributed 15 frames (containing 9 tampered frames) of watermarked ultrasound medical images to 4 cores, respectively. The main difference between (a) and (b) was that the tampered frames were organized in different order. The elapsed time for (a) is 5 seconds lesser than (b). It is because (a) has an even distribution of tampered frames and hence fairer workloads among 4 cores as compared to (b).

## 8. Conclusion and Future Work

The approach used to overcome the performance constraint in sequential watermarking process is by distributing ultrasound frames over multiple cores. The performance constraint for sequential watermarking process can be categorized into two problems: capacity and capability. The capacity problem occurred when the existing hardware and software are unable to perform the anticipated computations in an estimated time [18]. For example, it may not be feasible to conduct watermarking process on a large data size of DICOM files in any reasonable manner. Therefore, to run the entire computation may become impractical even though the existing hardware and software are capable of performing the watermarking process as required. The actual physical constraint, such as the processor speeds or total memory on a system, may cause the problem of capability, in which it will restrict the amount of watermarking processes performed. System upgrades may be a solution for the problem concern, but it is bounded by technology and cost constraint. In this case, the sequential watermarking problem may be partitioned into smaller and manageable parts that can be performed in parallel. Parallel computing with MATLAB is the simplest approach to leveraging multicores processor. However, the maximum number of parallel threads cannot exceed the number of cores available on the system. The performance gain obtained by using multiple cores on a single system is also limited and varies depending on the specific computation and the data size [9]. The proposed parallel watermarking scheme required little effort to separate the ultrasound medical images into a number of parallel tasks due to the little dependency (or communication) between those parallel tasks and achieved a speedup factor that is almost equivalent to the number of cores; for example, quad cores microprocessor will result in a speedup factor of approximately four, which means parallel watermarking process is approximately four times faster than sequential mode in quad cores microprocessor. Hence it could be classified as “embarrassingly parallel problems,” a phrase that comments on the ease of parallelizing such applications and the fact that it would be embarrassing for the programmer or compiler to not take advantage of such an obvious opportunity to improve performance [19]. The performance of proposed parallel digital watermarking scheme could be further improved by using graphics processing unit (GPU). Both CPU and GPU could run thousands of threads concurrently, but GPU will have a better performance than CPU due to its larger number of cores possessed relative to CPU; the difference in theoretical performance can differ by a factor ten in favour of the GPU; therefore a parallel digital watermarking operation on GPU is recommended. In many cases a hybrid CPU-GPU implementation yields the best performance. A good example is image registration algorithms, where the GPU can be used to calculate a chosen similarity measure in parallel, while the CPU can run a serial optimization algorithm. So et al. made a comparison between CPUs and GPUs for ultrasound systems, in terms of power efficiency and cost effectiveness. The conclusion was that a hybrid CPU-GPU system performed best [20]. The adoption of either GPU or hybrid CPU-GPU is largely dependent on the parallel adaption in an algorithm; an algorithm that exhibited “embarrassingly parallel problem” will be suitably used in GPU whereas hybrid CPU-GPU is suitably applied in an algorithm that exhibited “fine-grained parallelism.” For future work, the proposed method could be applied on Magnetic Resonance Images (MRI) where the ROI could be classified by using weighted-type fractional Fourier transform approach [21] prior to watermarking process. Since the watermarking is pixel oriented, thus it could be also applied on nature images with lesser restriction on image fidelity requirement as compared to medical images.

## Figures and Tables

**Table 1:** Comparison between task parallelism and data parallelism.

Task parallelism	Data parallelism
It is a form of parallelization of computer code across multiple processors in parallel computing environments [22].	It is a form of parallelization of computing across multiple processors in parallel computing environments [23].

Task parallelism focuses on distributing execution processes (threads) across different parallel computing nodes [23].	Data parallelism focuses on distributing the data across different parallel computing nodes.

**Table 2:** Stage in life cycle of a job.

Job stage	Description
Pending	A job is created on the scheduler with the *createJob* function in client session of Parallel Computing Toolbox software. The job's first state is pending. This is when the job was defined by adding tasks to it.

Queued	When the submit function is executed on a job, the MJS or scheduler places the job in the queue, and the job's state is queued. The scheduler executes jobs in the queue in the sequence in which they are submitted, all jobs moving up the queue as the jobs before them are finished. The sequence of the jobs in the queue can be changed with the *promote* and *demote* functions.

Running	When a job reaches the top of the queue, the scheduler distributes the job's tasks to worker sessions for evaluation. The job's state is now running. If more workers are available than are required for a job's tasks, the scheduler begins executing the next job. In this way, there can be more than one job running at a time.

Finished	When all of a job's tasks have been evaluated, the job is moved to the finished state. At this time, the results can be retrieved from all the tasks in the job with the function *fetchOutputs*.

Failed	When using a third-party scheduler, a job might fail if the scheduler encounters an error when attempting to execute its commands or access necessary files.

Deleted	When a job's data has been removed from its data location or from the MJS with the *delete* function, the state of the job in the client is deleted. This state is available only as long as the job object remains in the client.

**Table 3:** The relationship between parallel watermarking embedding and authentication process.

Parallel watermarking	Input file	Process applied on each task	Output file
Embedding process	Ultrasound (US) multiframe medical images/raw file	Watermarking embedding process	Watermarked US multiframe medical images

Authentication process	Tampered watermarked US multiframe medical images	Watermarking authentication process	Recovered US medical images and a message of tampered frame numbers

**Table 4:** Ultrasound medical images samples properties.

Ultrasound medical images	Image dimension in pixels	Bits per pixel	Number of frames
Ultraound_Sample_1.dcm	640 × 480	8	30
Ultraound_Sample_2.dcm	640 × 480	8	15
Ultraound_Sample_3.dcm	640 × 476	8	15

**Table:** (a) Watermarking embedding process on Ultrasound_Sample_1.dcm

Ultrasound frame number	Sequential		Parallel	
	MSE	PSNR	MSE	PSNR
1	1.0444	47.942	1.0444	47.942
2	1.0119	48.079	1.0119	48.079
3	1.0241	48.027	1.0241	48.027
4	1.0200	48.045	1.0200	48.045
5	0.9827	48.207	0.9827	48.207
6	1.0055	48.107	1.0055	48.107
7	1.0240	48.028	1.0240	48.028
8	0.99058	18.172	0.99058	18.172
9	0.99704	48.144	0.99704	48.144
10	1.0733	47.824	1.0733	47.824
*11*	*1.0224*	*48.035*	*1.0178*	*48.054*
12	1.0218	48.037	1.0218	48.037
13	1.0732	47.824	1.0732	47.824
14	1.0224	48.035	1.0224	48.035
15	1.0216	48.038	1.0216	48.038
16	1.0796	47.798	1.0796	47.798
17	1.0237	48.029	1.0237	48.029
18	1.0051	48.109	1.0051	48.109
*19*	*1.0175*	*48.055*	*1.0393*	*47.963*
20	0.97341	48.248	0.97341	48.248
21	0.99134	48.169	0.99134	48.169
22	1.0067	48.102	1.0067	48.102
23	0.97568	48.238	0.97568	48.238
24	0.99032	48.173	0.99032	48.173
25	1.0008	48.127	1.0008	48.127
26	0.97283	48.25	0.97283	48.25
27	0.98178	48.211	0.98178	48.211
28	1.0029	48.118	1.0029	48.118
29	0.97323	48.249	0.97323	48.249
30	0.99299	48.161	0.99299	48.161

**Table:** (b) Watermarking embedding process on Ultrasound_Sample_2.dcm

Ultrasound frame number	Sequential		Parallel	
	MSE	PSNR	MSE	PSNR
1	1.0338	47.986	1.0338	47.986
2	1.0329	47.99	1.0329	47.99
3	1.0491	47.923	1.0491	47.923
4	1.0506	47.916	1.0506	47.916
*5*	*1.0465*	*47.934*	*1.0597*	*47.879*
6	1.0616	47.81	1.0616	47.81
7	1.0502	47.918	1.0502	47.918
8	1.0458	47.936	1.0458	47.936
*9*	*1.0621*	*47.869*	*1.0595*	*47.88*
10	1.0632	47.865	1.0632	47.865
11	1.0611	47.873	1.0611	47.873
12	0.93946	48.402	0.93946	48.402
*13*	*1.0658*	*47.854*	*1.0591*	*47.882*
14	1.055	47.898	1.055	47.898
15	0.93955	48.402	0.93955	48.402

**Table:** (c) Watermarking embedding process on Ultrasound_Sample_3.dcm

Ultrasound frame number	Sequential		Parallel	
	MSE	PSNR	MSE	PSNR
1	0.86921	48.74	0.86921	48.74
2	0.87713	48.7	0.87713	48.7
3	0.88358	48.668	0.88358	48.668
4	0.9003	48.587	0.9003	48.587
5	0.90273	48.575	0.90273	48.575
6	0.91967	48.494	0.91967	48.494
7	0.91198	48.531	0.91198	48.531
8	0.9108	48.537	0.9108	48.537
9	0.91355	48.523	0.91355	48.523
10	0.93258	48.434	0.93258	48.434
11	0.9548	48.332	0.9548	48.332
12	0.95307	48.34	0.95307	48.34
13	0.96239	48.297	0.96239	48.297
14	0.93856	48.406	0.93856	48.406
15	0.92716	48.459	0.92716	48.459

**Table 6:** Sequential versus parallel watermarking embedding process in elapsed time.

Input file (ultrasound medical images)	Number of frames	Elapsed time in watermarking embedding process (seconds)		Speedup	Output file (watermarked ultrasound medical images)
		Sequential	Parallel		
Ultraound_Sample_1	30	738.70	38.30	19.29	Watermarked_US1
Ultraound_Sample_2	15	336.40	23.80	14.13	Watermarked_US2
Ultraound_Sample_3	15	332.60	22.80	14.59	Watermarked_US3

**Table 7:** Sequential versus parallel watermarking authentication process in elapsed time.

Input file (watermarked ultrasound medical images that have been fully tampered)	Tampered frame/total frame	Elapsed time in watermarking authentication process (seconds)		Speedup	Output file (tampered ultrasound file recovered as original ultrasound file)
		Sequential	Parallel		
Tampered_Watermarked_US1	30/30	597.1	164.2	3.64	Recovered_US1.dcm
Tampered_Watermarked_US2	15/15	270.0	92.6	2.92	Recovered_US2.dcm
^*∗*^Tampered_Watermarked_US3_a	15/15	241.2	73.3	3.29	Recovered_US3_a.dcm
^*∗*^Tampered_Watermarked_US3_b	15/15	259.1	71.0	3.64	Recovered_US3_b.dcm

^*∗*^Tampered_Watermarked_US3_a and Tampered_watermarked_US3_b are from the same source but have tampered differently.

**Table:** (a) Overall elapsed time taken in watermarking process on Ultrasound_Sample_1.dcm

Watermarking process	Sequential	Parallel	Speedup
Embedding	738.70	38.30	19.29
Authentication	597.10	164.20	3.64
Overall time taken	1335.80	202.50	6.60

**Table:** (b) Overall elapsed time taken in watermarking process on Ultrasound_Sample_2.dcm

Watermarking process	Sequential	Parallel	Speedup
Embedding	336.40	23.80	14.13
Authentication	270.00	92.60	2.92
Overall time taken	606.40	116.40	5.21

**Table:** (c) Overall elapsed time taken in watermarking process on Ultrasound_Sample_3.dcm

Watermarking process	Sequential	Parallel	Speedup
Embedding	332.60	22.80	14.59
Authentication	241.2	73.3	3.29
Overall time taken	573.8	96.1	5.97

**Table 9:** A table of comparison on elapsed time of watermarking authentication process on different sets of tampered frames in watermarked ultrasound medical images.

Tampered watermarked US images	Elapsed time for watermarking authentication process					
	Seq	D	R	Par	D	R
Tampered_0_frames	18.2	Yes	Yes	23.1	Yes	Yes
Tampered_3_frames	56.9	Yes	Yes	35.3	Yes	Yes
Tampered_6_frames	97.0	Yes	Yes	39.1	Yes	Yes
Tampered_9_frames	139.9	Yes	Yes	53.8	Yes	Yes
Tampered_12_frames	241.6	Yes	Yes	58.2	Yes	Yes
Tampered_15_frames	259.1	Yes	Yes	71.0	Yes	Yes

Seq: sequential version (seconds).

Par: parallel version (seconds).

D: able to detect and display the tampered frame number?

R: able to recover to its original form after authentication process?
